# The mathematics of multiple lockdowns

**DOI:** 10.1038/s41598-021-87556-6

**Published:** 2021-04-13

**Authors:** Antonio Scala

**Affiliations:** 1 CNR-ISC, Applico Lab, 00185 Rome, Italy; 2 Big Data in Health Society, Rome, Italy; 3 Gubkin Russian State University of Oil and Gas, Moscow, Russia

**Keywords:** Complexity, Statistical physics, thermodynamics and nonlinear dynamics

## Abstract

While vaccination is the optimal response to an epidemic, recent events have obliged us to explore new strategies for containing worldwide epidemics, like lockdown strategies, where the contacts among the population are strongly reduced in order to slow down the propagation of the infection. By analyzing a classical epidemic model, we explore the impact of lockdown strategies on the evolution of an epidemic. We show that repeated lockdowns have a beneficial effect, reducing the final size of the infection, and that they represent a possible support strategy to vaccination policies.

## Introduction

Mathematical models of epidemics help us understand how infectious diseases spread, and are useful to create scenarios on the likely outcomes of an epidemic, evaluating the effectiveness of public health interventions. In particular, such models help us to estimate important epidemiological parameters like the potential growth rate of an epidemic (the so-called basic reproduction number $\mathscr {R}_0$), the total fraction of people that will get infected (i.e. the final size $r_\infty$ of an epidemic), or the fraction of people to vaccinate in order to stop the epidemic (i.e. the herd immunity level $r^*$ ).

The actual COVID 19 pandemic has placed an heavy burden on health systems and economies, calling for more coordinated actions^1, 2^. Following the spread of COVID19, many countries had no choice but to issue stay-at-home orders and other non-pharmacological measures to buy time while a vaccine was developed. Such measures, often referred to as “lockdowns”, have never been applied before on such a large scale and their consequences are still subject of investigation. In this paper, by analyzing a classical epidemic model, we explore the impact of lockdown strategies on the evolution of an epidemic and show how multiple lockdowns could reduce the final size of the infection, thus representing a possible support strategy to vaccination policies when vaccines are either scarce or not yet available.

## Results

Among the models for epidemics, *SIR* models are often used for their simplicity. In such models, the population is divided into groups called compartments corresponding to different stages of an infection. In particular, S corresponds to susceptible individuals (i.e. people who can develop the disease), I to infectious (i.e. people who have developed the disease and can infect others) and R to recovered individuals. *SEIR* models are also often used; they are derived from *SIR* by introducing an extra class E corresponding to exposed individuals who have contracted the disease but are not yet infectious. We will indicate as “infected individuals” either the class *I* of the SIR model or the joint classes $E+I$ of the SEIR model. Introducing additional compartments can help to take account of other events like loss of immunity, births, deaths, healthy carriers^3^. We will indicate with lowercase letters (i.e. *s*, *i*, *e*, *r*) the fractions of individuals in a given class. For *S*(*E*)*IR* models, recovered individuals are considered to be immune to the disease; hence, a vaccination strategy aims to enlarge the fraction *r* of immune individuals beyond the herd immunity threshold $r^*$ ("Methods" section).

**Figure 1:**
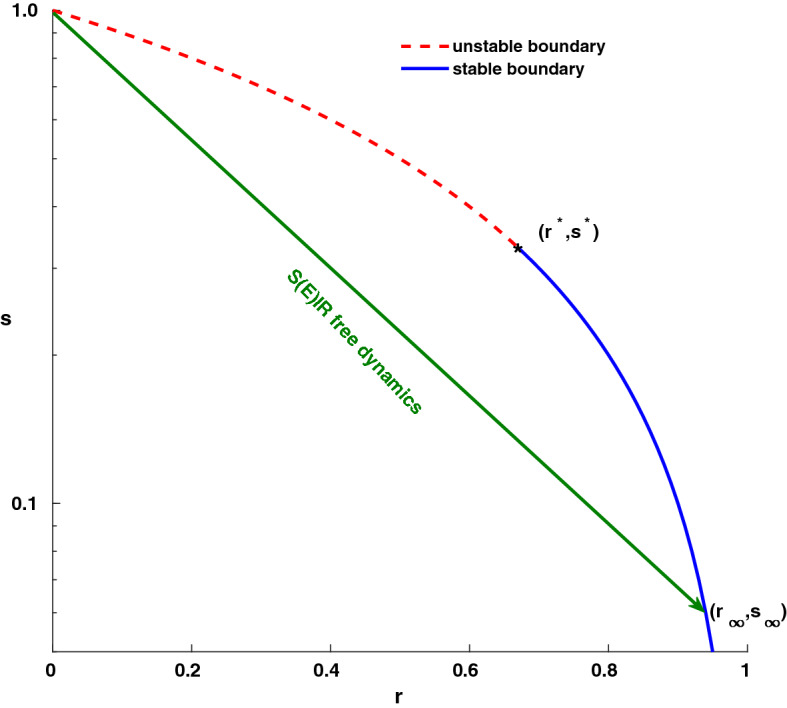
Unconstrained dynamics of *S*(*E*)*IR* epidemics. Horizontal axis: fraction *r* of recovered (immune) individuals; vertical axis: fraction *s* of susceptibles (logarithmic scale). The thick concave curve represents the “end of epidemic” boundary where no infectious individuals are present; the red dashed part of the curve represents unstable points, while the stable boundary is in blue. The thick green line (a straight line of slope $-\mathscr {R}_0$ , see Eq. (3) represents an epidemic outburst from the initial state $r=0$, $s=1$ where all individuals are susceptible; the arrow indicates the direction in which the epidemic evolves with time. The epidemic stops when reaching the “end of epidemic” boundary at the final size $r_\infty$; in general, $r_\infty >r^*$ (see "Methods" section). In the figure, $\mathscr {R}_0=3$ and the unconstrained epidemic reaches $r_\infty =94\%$, a value well beyond the herd immunity threshold $r^*=67\%$. Vaccination—if available—is a much better option than letting the epidemic unconstrained.

Let’s consider the case of a newborn epidemic: its evolution in the $r-\ln s$ plane corresponds to a straight line (Eq. 3) that starts from a population of fully susceptible individuals (i.e. $s_0=1,r_0=0$); the epidemic ends when such curve intersect the “end of epidemic boundary” $\ln s^\Omega$ (Eq. 4). As shown in "Methods" section, the intersection point $P_\infty = (r_\infty , s_\infty )$ is stable (i.e. no epidemic outburst are possible) since the final size $r_\infty$ of the epidemic is strictly greater than the herd immunity threshold $r^* =1-\mathscr {R}_0^{-1}$. To give a flavour of the difference between letting an epidemic freely evolve against vaccinating a population, let’s consider the case $\mathscr {R}_0=3$—a value that has been estimated for COVID19 in France^4^ ; with such a basic reproduction number, at the end of the epidemic a percentage of $r_\infty =94\%$ individuals would have been infected, while only $r^* =67\%$ are needed to reach herd immunity. Thus, vaccination is a much better option, since letting the epidemic evolve freely always go beyond the epidemic threshold (see Fig. 1).

**Figure 2:**
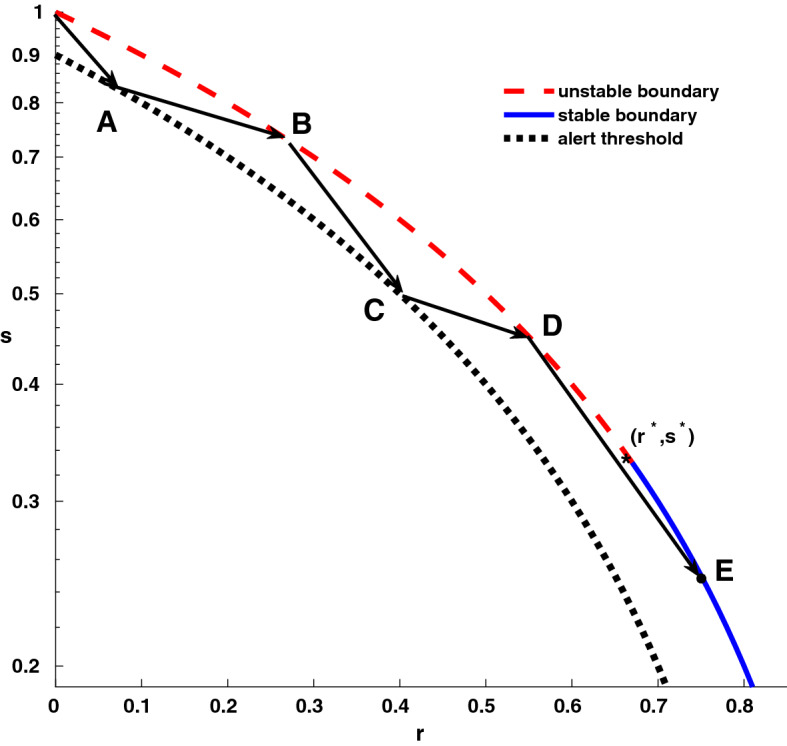
Schematic dynamics of a lockdown strategy for a *S*(*E*)*IR* epidemic. Horizontal axis: fraction *r* of recovered (immune) individuals; vertical axis: fraction *s* of susceptibles (logarithmic scale). The thick concave curve represents the “end of epidemic” boundary where no infectious individuals are present; the red dashed part of the curve represents unstable points, while the stable boundary is in blue. The black dotted concave curve corresponds to a fraction of $10\%$ of the population being infected. In the picture, we show a simple strategy that contains an epidemic with basic reproduction number $\mathscr {R}_0=3$ by lockdowns that reduce the reproduction number to $\mathscr {R}_0^{lock}=0.7$ whenever the fraction of infectious individuals hits the alert threshold $\theta =10\%$. The evolution of unconstrained and “locked down” epidemics are represented by straight lines ending with arrows. Lockdowns corresponds to the segments $\overline{AB}$ and $\overline{CD}$; since, for the parameter chosen, their ending point *B*, *D* belong to the unstable boundary (i.e. $r<r^*$), the epidemic will start again when the lockdown is lifted. The process of unconstrained epidemics and lockdown interventions repeats again until a final point beyond herd immunity is reached. For the chosen parameters, the epidemic stops when hitting the stable boundary at the point *E* where the fraction of recovered individuals is $r_E=76\%$ of the population, less than the size $r_\infty =94\%$ of the unconstrained epidemic (see Fig. 1), but still higher than the herd immunity threshold $r^*=67\%$ . Again, vaccination—if available—is a better option.

What happens if no vaccine is available yet, or if there is an insufficient amount to reach at least the epidemic threshold? A possible solution is to mitigate the epidemic spread by reducing social contacts among the people via so-called “lockdowns”. Lockdown strategies are one of the possible non-pharmaceutical interventions that try to minimize the number of infected while buying time for the production of vaccines; in the case of the recent COVID19 pandemic, this approach has been forced by the necessity of not overloading intensive care units. Trying to minimize the number of infections leads to a final state at the end of a lockdown that could be unstable due to the lack of a sufficient number of recovered individuals. In such a case, the population will be susceptible to a new outbreak when lifting the lockdown measure. Thus, the epidemic would eventually spread again and a new lockdown would have to be enforced until either the final state becomes stable or a vaccination campaign takes in. To illustrate such a mechanism, we depict in Fig. 2 an idealised case where, for an epidemic with basic reproduction number $\mathscr {R}_0=3$, the lockdown strategy takes in by reducing the reproduction number to $\mathscr {R}_0^{lock}=0.7$ (a value in the range observed for COVID19 non-pharmacological interventions^5^) whenever the number of infectious individuals hits the $10\%$ of the population (here we are assuming that, as in COVID19, only a small fraction of infectious individuals needs intensive care). With these parameters, if no vaccine is found, the epidemic stops after 2 lockdowns and 3 outbreaks, when the fraction of recovered individuals reaches the $76\%$ of the population (see Fig. 2): a value that is less than the size of epidemics $r_\infty =94\%$ (i.e. the one reached in absence of any intervention), but bigger than the epidemic threshold $r^* =67\%$. In general, since Eq. (3) intersects the “end of epidemic” boundary (Eq. 4) at fraction of recovered that is a strictly increasing function of the epidemic starting point $r_0$, multiple lockdowns will always stop with a fraction of recovered individuals less than the final size $r_\infty$ but greater than the herd immunity threshold $r^*$.

As shown in “Methods” section, once the lockdown hits the “end of epidemic” boundary, an epidemic can start if and only if $\mathscr {R}_0>(1-r)^{-1}$; i.e., the critical value of the basic reproduction number becomes bigger than one. As an example, at the point B of Fig. 2 (i.e. after the first lockdown), an epidemic can start only if the basic reproduction number is $>1.31$, while at the point D (i.e. after the second lockdown) it can start only if the basic reproduction number is $>1.92$. Thus, if we consider the reduction of social contacts in the scenario depicted by Fig. 2, we see that while at the beginning of the epidemic a reduction of $67\%$ of social contact is needed, after the first lockdown (point B) a reduction by $57\%$ suffices, and after the second lockdown (point D) is sufficient an even milder reduction by $37\%$.

## Discussion

Lockdowns are novel measures to contain large scale epidemics in absence of—and waiting for—vaccines. As pointed out by the WHO, “these measures can have a profound negative impact on individuals, communities, and societies by bringing social and economic life to a near stop” (Coronavirus disease (COVID-19): Herd immunity, lockdowns and COVID-19, accessed 21 Jan 2021). In fact, it has been shown that mobility restrictions associated with lockdowns not only have economic and social consequences^6^, but also impact on international trade^7^: already in May 2020, a World Bank Policy Research Working Paper suggested that non-pharmacological interventions had led to a decline of about $10\%$ in the economic activity across Europe and Asia^8^. Non-pharmacological interventions are proving extremely costly also in terms of mental health, both for health professionals^9^ and for the general population^10^, possibly with long term consequences^11^. Thus, it is important to reach a general understanding of lockdowns’ dynamics—especially when lockdowns recur more than once. To such an aim, coordinated inter-disciplinary approaches building up on data-driven scientific communities are welcome^12^.

One of the most controversial issues of the COVID19 pandemic has been the idea of reaching herd immunity by letting the epidemic evolve. Even assuming that recovered individuals are immune to reinfection (an assumption that is strongly challenged by medical data^13^, which suggest that even recovered individuals can be infectious^14^), the toll on the population would be too high due to the high mortality rate: for COVID-19 herd immunity by infection is not an option^15^, but a false promise^16^. Moreover, when the fraction of recovered individuals reaches to the herd immunity level in an unconstrained epidemic, the number of infected individuals is not zero; hence, without restrictive measures and/or vaccination policies, the final toll of an epidemic will be always higher than what is needed, since it maximizes the overshoot^17^ beyond the herd immunity (see Sect. 4). To such an end, the policies adopted in Sweden with respect to COVID-19 represent an enigma^18^.

While many numerical and data-driven investigations have explored the impact of lockdowns^19^, a general understanding of their consequences—especially when lockdowns recur more than once—is yet to be reached. The possibility that, due to the strong social contact restrictions, lockdowns would have to be re-enacted more than once, was clear since the beginning of the COVID19 epidemic^20^: in fact, at the end of July 2020 it had already been assessed that no country had yet seen infection rates sufficient to prevent a second wave of transmission^21^. We have shown that repeated lockdowns decrease the final toll of an epidemic from the size of the “free“ epidemic $r_\infty$ to a value nearer to the herd immunity level $r^*$; in general, repeated lockdowns will end with a total fraction of infected individuals between these two values.

An important issue to be addressed is the heterogeneity of the population: it has been shown that taking into account heterogeneity can both enhance the effectiveness of lockdown policies^20^ and positively influence the herd immunity level^22^; to such an aim, it is important to infer high-resolution human mixing patterns for disease modelling^23^. However, although heterogeneity can enhance the severity of an epidemic due to super-spreaders^24^, it not expected to change qualitatively the scenarios depicted by *S*(*E*)*IR* models but in the case of extreme heterogeneity^25^.

Finally, we have shown that, at the end of each lockdown, the critical value of the basic reproduction number above which an epidemic can start increases, bringing thus the population in a state where milder and milder measures are needed to prevent an outbreak. It has been suggested that the behavioural response to an outbreak of a severe disease can induce the contact rates to decrease with time^26^; consistently, it has been observed that changes in the mixing patterns of a population during and after lockdown can decrease $\mathscr {R}_0$^27^. Thus, it could be the case that mild non-pharmacological interventions (like wearing masks or implementing network-based lockdowns^28, 29^ to avoid the pitfall of super-spreaders^25^) with a lower impact on the economy of a country (or on the mental health of its inhabitants) could be used to contain an epidemic while waiting for the vaccine or while implementing challenging mass vaccination policies^30^. In particular, in presence of limited but significant amount of vaccines, it would be even possible to use mild lockdown strategies to held the population above the lockdown herd immunity threshold (Eq. 6) until sufficient vaccines are produced.

## Methods

In terms of the fractions *s*,*i*,*r* of susceptible, infectious and recovered individuals, the *SIR* model is described by a set of deterministic differential equations:

1 $$\begin{aligned} {\begin{matrix} \partial _t s = - \beta s i \\ \partial _t i = \beta s i - \gamma i\\ \partial _t r = \gamma i \end{matrix}} \end{aligned}$$

where the transmission coefficient $\beta$ is the rate at which a susceptible individual becomes infected upon meeting an infectious individual, and $\gamma$ is the rate at which infectious individuals are removed from the infection cycle. In the *SEIR* model

2 $$\begin{aligned} {\begin{matrix} \partial _t s = - \beta s i \\ \partial _t e = \beta s i - \mu e \\ \partial _t i = \mu e - \gamma i \\ \partial _t r = \gamma i \end{matrix}} \end{aligned}$$

the dynamics of the exposed fraction *e* is introduced; here $\mu$ is the rate at which exposed individuals become infectious.

Since for both models $\partial _t s = -(\beta /\gamma ) s \partial _t r$, we have that $\partial _t \ln s = -\mathscr {R}_0 \partial _t r$, where $\mathscr {R}_0=\beta /\gamma$ is the so-called basic reproduction number. Thus, the evolution of the *s* can be expressed just in terms of *r* as

3 $$\begin{aligned} \ln s = \ln s_0 - \mathscr {R}_0\cdot \left( r-r_0\right) \end{aligned}$$

showing that *S*(*E*)*IR* epidemics are straight lines of slope $-\mathscr {R}_0$ in the $r-\ln s$ plane. The time dependence of such trajectories can be recovered by inverting parametric solutions of the model^31^.

The analysis of phenomenological models based on the renormalization group approach applied to recurrent COVID19 waves^32^ have shown that time dependent parameters are useful in describing the ongoing pandemics^33^; however, variable parameters will not qualitatively change the framework of stability analysis we are going to deploy.

### Herd immunity

If we indicate with *a* the fraction of infected individuals (i.e. $a=i$ in SIR and $a=i+e$ in SEIR), from Eqs. (1) and (2) derives that in *S*(*E*)*IR* models $\partial _t a \propto \mathscr {R}_0 s -1$, so that for $s^*<\mathscr {R}_0^{-1}$ the number of infected individuals decreases without creating an epidemic outbreak. The threshold $r^*=1-s^*$ is the so-called herd immunity threshold; vaccination policies aim precisely to immunize a fraction of the population greater than $r^*$. Notice that, during an epidemic, the fraction of infected individuals reaches the maximum at $s=s^*$ since at this point $\partial _t a =0$.

An epidemic burst ends when the fraction of infectious is zero, corresponding to the condition $s+r=1$. Thus, in the $r-\ln s$ plane, the final size of an epidemic corresponds to the intersection of the straight line described by (Eq. 3) with the “end of epidemic” boundary curve

4 $$\begin{aligned} \ln s^\Omega = \ln \left( 1-r\right) \end{aligned}$$

that is a concave, strictly decreasing function of *r* since slope $\partial _r \ln s^\Omega =-1/(1-r)$ is also a strictly decreasing function of *r*. However, such points are stable only if $r>r^*$.

An epidemic ends at a point $(s_{end},r_{end})$ that is the rightmost intersection of Eq. (3) with the boundary; since $r_{end}$ accounts for the fraction of people that have been infected, it is also called the epidemic size^34^. The point $P^*=(s^*,r^*)$ separates the unstable from the unstable part of the “end of epidemic” boundary; at this point the slope is $-\mathscr {R}_0$. Since $\ln s^\Omega$ is strictly concave, $P^*$ is the farthest point to any straight line of slope $-\mathscr {R}_0$ in the $\ln s-r$ plane, i.e. from any unconstrained dynamics (see Eq. 3). Thus, any unconstrained dynamics will end in a point with $r>r^*$ that is therefore stable.

Among all the free dynamics starting from the unstable part of the “end of epidemic” boundary (i.e. $r<r^*$), the one starting from the “natural” initial state $r_0=0$, $s_0=1$ (i.e. all individuals are susceptible) ends at the point $(s_\infty ,r_\infty )$ with the highest fraction of recovered individuals, i.e. it is the epidemic that accounts for the highest epidemic size.

### Lockdowns

While vaccination is the optimal response to an epidemic, recent events have obliged to explore new strategies for containing worldwide epidemics via lockdown strategies, where the contacts among the population are strongly reduced in order to slow down the propagation of the infection. Lockdown strategies are non-pharmaceutical interventions exploiting the fact that the transmission coefficient $\beta$ can be thought as the product $C \lambda$ of a contact rate *C* (related to social habits and interactions) times a disease-dependent transmission probability $\lambda$. A lockdown strategy aims to decrease the contact rates, resulting in a *S*(*E*)*IR* dynamic with a reduced basic reproduction number $\mathscr {R}_0^{lock} < \mathscr {R}_0$. A natural measure of the lockdown strength is the parameter $\delta ^{lock}$

5 $$\begin{aligned} \delta ^{lock} = 1-\mathscr {R}_0^{lock}/\mathscr {R}_0 \end{aligned}$$

that is can be interpreted as the decrease of contact rates needed to reach a given lockdown level.

For *S*(*E*)*IR* dynamics, a lockdown corresponds to a straight line of slope $-\mathscr {R}^{lock}_0$ in the $r-\ln s$ plane. Following the same reasoning of "Herd immunity" section, such dynamics will intersect the boundary beyond a point $r^*_{lock}=1-1/\mathscr {R}_0^{lock}$. Since $r^*_{lock}<r^*$, a lockdown dynamics can end with a fraction $r_{end}$ of immune individuals that is unstable respect the unconstrained epidemics, i.e. $r^*_{lock}\le r_{end} \le r^*$. In such a case, releasing the lockdown can result in a new epidemic outburst.

A vaccination policy aims to bring the fraction of recovered individuals *above* the herd immunity threshold $r^*$ by inoculating vaccines and avoiding the people experiencing a dangerous disease course. However, $r^*$ normally corresponds to an high fraction of the population: for new-born epidemics, it can be the case that it is not possible to produce enough vaccine before the epidemic ends. Let’s assume that, by non-pharmaceutical interventions, epidemic has been dampened out and that an amount of vaccines of efficacy $\epsilon <1$ useful to immunize a fraction *v* of the population has been produced; however, let’s also assume that the vaccines produced are not sufficient to reach herd immunity threshold since $\epsilon v < r^*$. It it possible to imagine to introduce a lockdown that reduces the herd immunity threshold to available vaccination capabilities, i.e. $r^*_{lock}=\epsilon v$? This goal can be accomplished by noticing that for a lockdown implements a given herd immunity threshold $r^*_{lock}$ if $\mathscr {R}_0^{lock} =1/(1-r^*_{lock})$; thus, indicating with $\mathscr {R}_0^{vax}$ the lockdown level corresponding to a vaccine immunization of a fraction *v* of the population, we have that $\mathscr {R}_0^{vax} =1/(1 - \epsilon v)$. Thus, the strength $\delta ^{vax}$ of a lockdown to reach herd immunity in presence of partial vaccination is

6 $$\begin{aligned} \delta ^{vax} =1-\frac{1-r^*}{1 - \epsilon v}. \end{aligned}$$
